# Iterative Precise Conductivity Measurement with IDEs

**DOI:** 10.3390/s150512080

**Published:** 2015-05-22

**Authors:** Jaromír Hubálek

**Affiliations:** Centre of Sensors, Information and Communication Systems, Faculty of Electrical Engineering and Communication, Technicka 3058/10, Brno 616 00, Czech Republic; E-Mail: hubalek@feec.vutbr.cz; Tel.: +420-54114-6195; Fax: +420-54114-6298

**Keywords:** IDEs, electrolytic conductance, specific conductivity, iterative measurement, cell constant correction

## Abstract

The paper presents a new approach in the field of precise electrolytic conductivity measurements with planar thin- and thick-film electrodes. This novel measuring method was developed for measurement with comb-like electrodes called interdigitated electrodes (IDEs). Correction characteristics over a wide range of specific conductivities were determined from an interface impedance characterization of the thick-film IDEs. The local maximum of the capacitive part of the interface impedance is used for corrections to get linear responses. The measuring frequency was determined at a wide range of measured conductivity. An iteration mode of measurements was suggested to precisely measure the conductivity at the right frequency in order to achieve a highly accurate response. The method takes precise conductivity measurements in concentration ranges from 10^−6^ to 1 M without electrode cell replacement.

## 1. Introduction

Using comb-like structures of electrodes (interdigitated electrodes—IDEs) with different sizes is still under intensive research as many published articles in the areas of chemical sensing or characterization [1,2,3,4,5,6] and biosensing [7,8,9,10,11,12,13] have shown this year.

**Figure 1:**
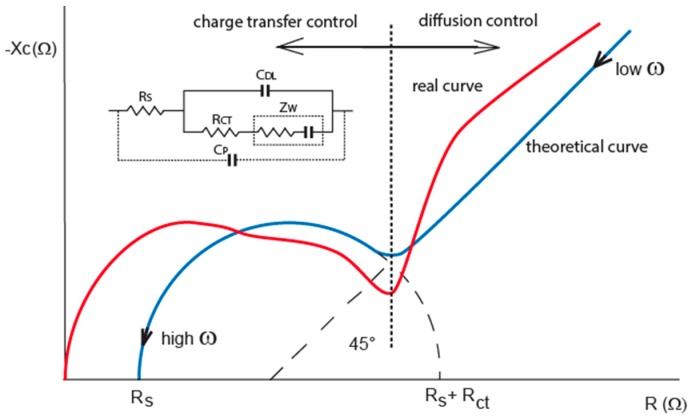
Nyquist plots of interface impedance, theoretical locus (blue curve) and real locus (red curve). The real locus shows parasitic capacitance overlapping double layer capacitance. Randles equivalent circuit is drawn in the inset of graph. Additional parasitic capacitance is involved for real behavior circuit of interdigitated electrodes (IDEs).

Electrochemical impedance behavior was already described by prof. Randles in the 40’s of the last century and the most used model is called Randles equivalent circuit as double layer capacitance (C_DL_) and charge transfer resistance (R_CT_) in parallel and solution resistance (R_S_) in series [14]. The behavior of the impedance model presents a semicircle curve at high frequencies in the complex plane [15]. The model was updated later by adding nonlinear behavior at low frequencies (diffusion phenomena) (Figure 1, blue line) [15,16]. The importance of the dependences of the parameters of the theoretical model are very well described in [17]. Solution resistance is the aim of conductometry and it depends directly on ion concentration. The double layer capacitance depends on the voltage and the diameter of ion atmosphere of the solvated ions, and it is indirectly related to the concentration of the solvated ions. Charge transfer resistance performs the charge exchange between ions and electrode through the double layer and it strongly depends on the overpotential of the electrode. The non-linear part of impedance model is controlled by diffusion. It is presented by derived resistance and capacitance in series called Warburg impedance (Z_W_) depending on the square root of frequency (linear slope with angle 45° in the Figure 1). The cell constant is determined by interelectrode distance *d* over a cross-sectional area *A*. Standard specific conductivity measurements use large electrodes in the cell to obtain huge double layer capacity leading to very small impedance already at low frequencies about 10^−2^ Hz. Then the R_CT_ with Z_W_ is eliminated from the measurement and the cell constant is actually determined by geometrical parameters because insignificant edge effects of the large electrodes do not bring any distortion. In the case of IDEs, this part of Randles circuit and their dependencies have to be included into consideration and it is also confirmed in the case of biosensors [18].

**Figure 2:**
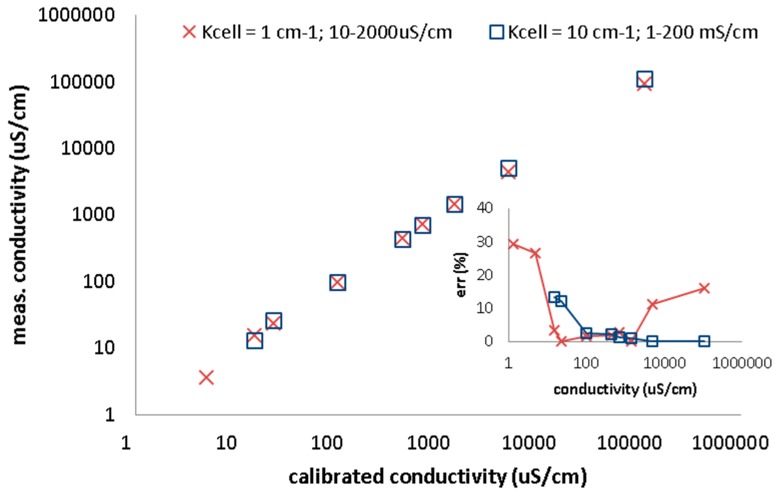
Conductivity measurements with CyberScan PC6500. Two electrodes with cell constant 1 and 10 cm^-1^ calibrated at two points. Measured conductivity *versus* calibrated samples are plotted. Inset of figure presets relative error of measured conductivity. The error is high out of the cell conductivity range.

Empirical experiences show that keeping AC amplitude below 100 mV can avoid harmonic distortion because no polarization of electrode occurs [19]. The electrode design and construction impact the measurement significantly. Therefore, the use of IDEs is determined by their arrangement and impedance behavior. Because the geometric sizes of IDEs cannot be used for the calculation of the cell constant, it is usually calibrated in a known solution. Standard conductometric electrodes are calibrated to a certain cell constant and they work at a certain range of measured conductivity to assure defined accuracy of measurement [20,21]. Out of this range the error increases as shown in data obtained from conductivity analyzer CyberScan PC6500 (Eutech Insruments, Landsmeer, The Netherlands) using two cells with two-point calibration (Figure 2). Therefore, the cell constant or its correction factor is determined by the calibration. Conductivity measurements usually use a constant frequency, *i.e.,* it is obvious the frequency is chosen according to Nyquist diagram to measure at the control of charge transfer resistance close to Rs with minimal reactance.

**Figure 3:**
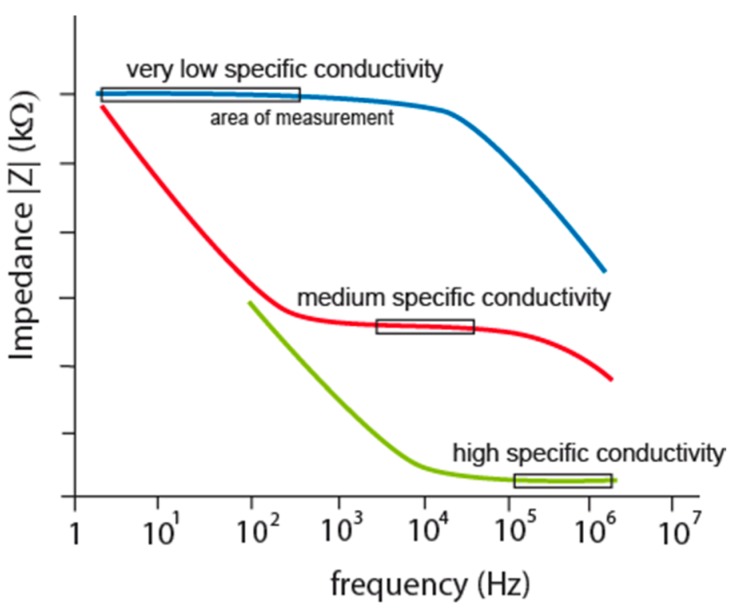
Impedance characteristics of IDEs according to different measured specific conductivity.

IDEs are usually fabricated using microtechnologies and mostly used for electrochemical impedance spectroscopy or conductometry of ions or biomolecules in electrolyte. A specific conductivity is calculated from measured conductivity between electrodes and the cell constant. The need of correction factor and optimization of design have been shown in several articles due to the planar form and small sizes of IDEs [22,23,24]. IDEs are designed to have small spacing *d* between the fingers and high cross-sectional area which increase current response to take the measurement above noise. This design increases parasitic capacitance of planar electrodes on any substrate leading to zero impedance at high frequencies [25]. It is clear that semi-circle locus of the double layer is impacted with this parasitic capacitance (Figure 1, red curve). Measuring frequency cannot be set at high frequencies because interface impedance is going through parasitic capacitance to zero value but it has to be set to frequency, where double layer and charge transfer resistance are most significant. Impedance characteristics in that case show the boundary: lower by double layer and upper by parasitic capacitance for measuring frequency determination, see Figure 3, red curve.

**Figure 4:**
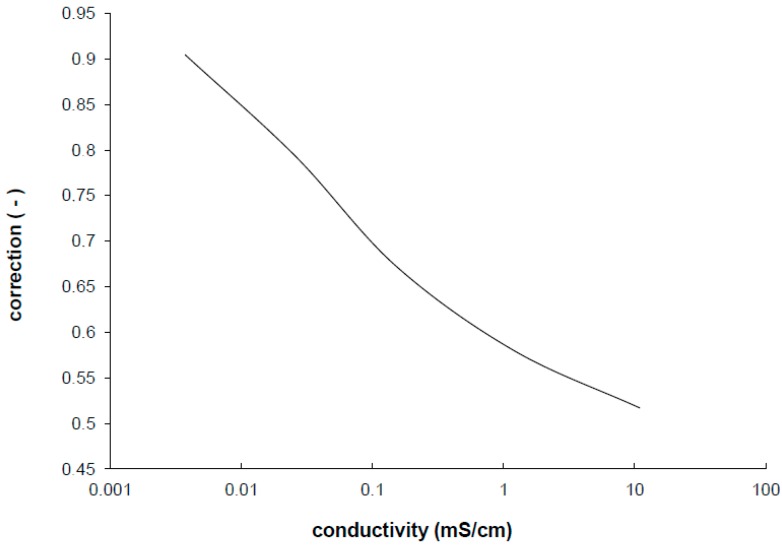
The correction factor of the cell constant experimentally determined from the measurement of the screen printed IDEs.

Empirical results confirm that for each application of each IDEs the measuring frequency has to be found. In addition, the impedance characteristics are significantly shifted to the double layer part or the diffusion part corresponding to the measured conductivity of ions in electrolyte. This shift results in stronger dependency of cell constant correction on the specific conductivity (Figure 4) than in the case of standard electrodes [25]. The measuring range of conductivities has to be very narrow to keep the correction factor very small and to avoid worsening the accuracy. Therefore, many different cell constants have to be used for measured range of conductivity to obtain wide range conductivity measurement with IDEs and good accuracy. This means that the range of specific conductivity to be measured with declared precision is much smaller in comparison with any standard electrodes.

The reason was discovered in current density between electrodes which push current flow in transversal direction from electrodes as the electrolyte concentration is diluting [25,26]. The path of the current can be imagined as semi-circles with increasing average distance between the electrodes. These impacts cause frequency characteristics shifts shown in Figure 3 by different colors for low, medium and high concentrated electrolytes as low, medium and high specific conductivity respectively. Because the flat parts are suitable for measurements (the area of measurement in Figure 3) there is no measuring frequency mutual for all three cases.

**Figure 5:**
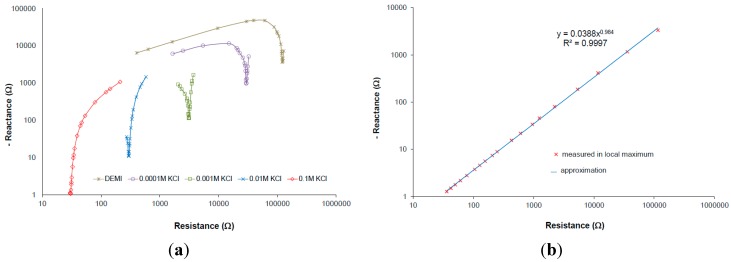
(**a**) Impedance in logarithmic Nyquist plot of different electrolyte concentrations measured by IDEs, parameters: 1 mm spacing, 1 mm electrode width, 7 mm length, six fingers; (**b**) Complex plain presenting impedance taken at local maximum for different conductivity/concentration of measured electrolyte. Reprinted from [25], Copyright (2015), with permission from Elsevier.

It was already presented on screen-printed IDEs that the measurements taken in local maximum of capacitive imaginary part (see Figure 5a: it is minimum in the plots because of negative y-axis) is close to linear dependency under logarithmic Nyquist plot. Measurement taken at this local maximum of capacitive imaginary at exact frequency can be called the frequency at local maxima of several samples with different conductivity (see Figure 5b) [25].

The measurements took in local maximum of an imaginary part of the interface impedance correspond very well with conductivity [25]. At this point, at the frequency at local maxima, the capacitive part of the impedance interface is very small and the resistance is between the solution resistance (R_S_) and the sum of R_S_ plus the charge transfer resistance (R_CT_). The R_CT_ does not change if the measuring voltage is smaller than 100 mV [16]. In this case measured resistance can be easily corrected within real R_S_. Therefore, we can express that

(1) Re(κ)=k(κ)⋅Rs

Where Re is the real part of impedance taken in maximum of interface impedance shown in Nyquist plot (Figure 5, minimal reactance), *k* is correction depending on the specific conductivity. The correction is determined by calibration and it can be related to the cell constant correction factor because it can be shown that it has the same curve line dependency on the conductivity in case of IDEs.

An uncorrected relative error of the interface impedance measurement can be determined at the minimum capacitive part by

(2) Δ≡|Z′measured−RsRs|=|Re(κ)−RsRs|=k(κ)−1

When correction k(κ) is used the error of the conductivity measurement will be significantly reduced and independent on measured conductivity.

In this work the iterative method of precise conductivity measurement by IDEs will be presented. The method is based on previous observed characteristics and dependences presented in [25]. How to obtain the precise conductivity measurement with IDEs at wide range of the electrolyte conductance will be shown in the results.

## 2. Experimental

The IDEs used for confirmation of this work were printed using standard screen printing technique. Platinum comb-like structure with five fingers of one electrode was printed by Pt paste (Dupont DP 9894, USA) on polished alumina substrate (96%). Non-electrode parts as leads were covered with overglaze paste (ESL 4026-GREEN, UK) [25].

An interface impedance characterization of the IDEs has been performed with a precise impedance analyzer (Agilent 4284) connected to PC as it was already presented in [25]. Automatic impedance spectrometry was taken using LabView. The IDEs, through testing, gives us a better insight to the behavior of this electrode shape.

The confirmation of the developed method was done using the same setup. Each step was performed manually to set new frequency for next measurement after calculation from last measured impedance values. The steps were repeated till the frequency was possible to setup due to the resolution of the analyzer frequency synthesizer.

## 3. Method Development

**Figure 6:**
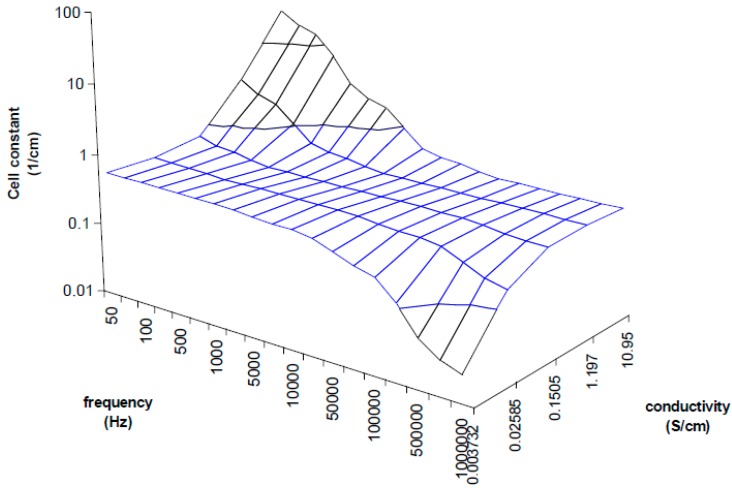
3D plot demonstrating cell constant deviation according to the measuring frequency and measured conductivity. Unaccepted deviations are detected at low conductivity measured at high frequencies and high conductivity measured at low frequencies.

A better view of the IDEs behavior were plotted onto a 3D plane as shown in Figure 6 in order to assist easier understanding of the cell constant dependency characteristics. The measurements were taken with known samples and the right cell constants were determined at different frequencies. The strong deviations are about more than one logarithmic decade of cell constant for very low or high frequencies, and they specify the correction factor necessity. In the range from 5 kHz to 10 KHz the cell constant seems to be constant when the frequency is increasing due to measured conductivity increases as well, therefore the plane is flat in these range borders (as shown in the Figure 6) which means low deviations for low or high concentrations, but still this deviation causes unacceptable error of measurement overcrossing 10%. Out of these range borders the cell constant can increase (at low frequencies) or decrease (at high frequencies) about two orders of cm^−1^. Moreover, another size of IDEs will have other characteristics. It is clear that the frequency has to be changed as the specific conductivity changes to get a very small deviation of the cell constant over the wide range of measurements.

Our experimental results confirm this behavior, where only values at the frequencies at local maxima were taken within the minimum of the reactance for the wide conductivity range of KCl solution [4]. The frequency at local maxima characteristics can be expressed in a complex plane as a power function of the real and imaginary part of the interface impedance (Figure 5b). The real part of the interface impedance has an approximately linear relation with frequency and conductivity. Extracted relations are expressed in Equations (3) and (4).

(3) Re=C1⋅f−α

(4) Re=C2⋅κ−β

The fraction α/β is approximately equal to 1. In our case of IDEs parameters α is 1.005 and β is 0.995, C_1_ is approx. 2.10^7^ and C_2_ is 253. It is clear that if the real part of the impedance depends on the frequency at local maxima and conductivity, the relation between both variables can be derived from Equations (3) and (4). It can be expressed by Equation (5).

(5) $$f={\left(\frac{C_{2}}{C_{1}}\right)}^{-\frac{1}{\alpha }}\cdot \kappa^{\frac{\beta }{\alpha }}=a\cdot \kappa +b$$

**Figure 7:**
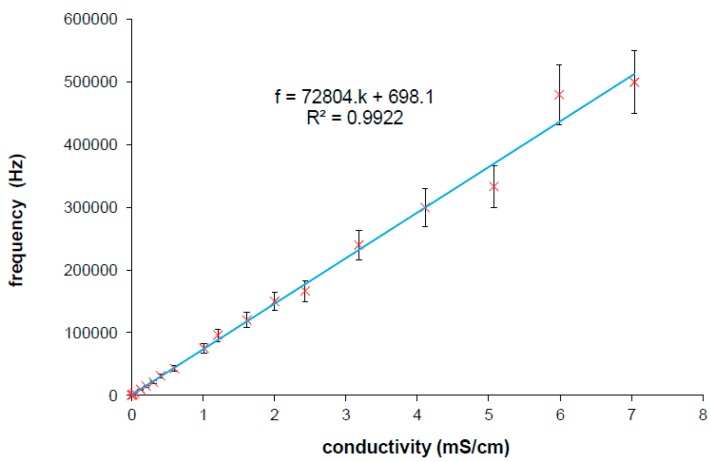
Linear dependence of frequency at local maxima on measured specific conductivity obtained from measuring various specific conductivities by IDEs [25]. Parameters for Equation (5) are determined from linear regression (inset of figure).

The constants a,b have to be determined experimentally for each IDEs. The characteristic is linear (it was published in [25]) and it can be understood as the correction characteristics which is expressed as the frequency at local maxima within the variable measured specific conductivity. The characteristics according to Equation (5) obtained from measured results is presented in Figure 7 which corresponds with imaginary line in 3D graph (Figure 6) linking left and right corner of the plane parts in the plot. The linear regression from the curve was used rather than from Equations (3) and (4) because of additive error. The intercept b is not equal to zero because of additive error of regression and ratio β/α unequal to 1. The Equation (5) will be finally used in iteration method for automatic measurements which enables the tuning of the measuring frequency according to specific conductivity resulting in suppressing strong cell constant dependence of IDEs.

As it was described above, the cell constant strongly depends on the measured conductivity in case of IDEs. Because the interface impedance is measured, the cell constant should be derived from this impedance taken at the frequency at local maxima. An extracted equation is

(6) $$K_{cell}=-c\cdot Ln(Z^{\prime }(f_{M}))+d$$

The constants *c*,*d* have to be determined experimentally, *f_M_* is measuring frequency at local maxima. When the influence of conductivity on the cell constant is very strong, the correction of the cell constant could be performed according to Equation (6) to increase the accuracy of measurement.

## 4. Results and Discussion

**Figure 8:**
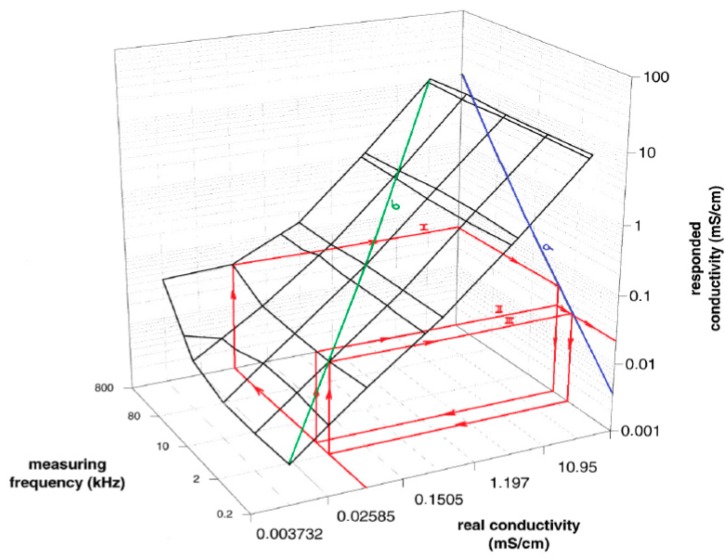
The 3D graph presents relation between real specific conductivity which is going to be determined, and responded specific conductivity, which differ according to the measuring frequency applied to IDEs. Demonstration of the iterative algorithm running in three steps of approximation to the measured real specific conductivity is represented by red arrows.

The measurement has to be taken at the frequency at local maxima corresponding to the measured conductivity value. It can be simply achieved with iterative algorithm as mentioned above. The responded conductivity is calculated from measured resistance at set frequency, using iterations according to Equation (5) until the difference between the last calculated conductivity and the new conductivity is close to zero. Because the measured conductivity is not known at the beginning of the measurement, subsequent approximations to the frequency at local maxima and to the corresponding conductivity have to be performed in a few repeated measuring steps. This iterative method of the measurement is demonstrated clearly in Figure 8.

The 3D plot demonstrates the measured and the responded conductivity at different frequencies at local maxima and the real conductivity of our planar electrodes (IDEs). The graph is going from the plot in Figure 7, it is the blue line (a) in the plot of Figure 8; the green line (b) is its 3D representation; 3D plane demonstrates impedance interface of IDEs behavior. If a solution with the conductivity value of 0.02585 mS/cm is measured at the start frequency of 800 kHz, the iteration steps go from the front of x-axis in compliance with red arrow to the back, where measuring frequency is 800 kHz. Then the steps continue in direction of arrows to determine the responded conductivity (I). In the second step (II), a new measuring frequency is extrapolated from the curve (a) to do a new measurement at frequency determined by Equation (5). Steps are repeated until the responded conductivity is equal to the measured real conductivity (step III in Figure 8). The method quickly finds the correct frequency at local maxima for a measured conductivity.

**Table 1:** Iterative steps provided by Agilent 4284A for two modes.

Mode 1						Mode 2		
**It.**	**Calcul. Freq (kHz)**	**Meas. Freq (kHz)**	**Re (kOhm)**	**Im (Ohm)**	**Conduct. (mS/cm)**	**It**	**Calcul. Freq (kHz)**	**Conduct. (mS/cm)**
1		1000	1.598	−589.2	0.1444	1		0.1482
2	11.243	11.3636	2.1375	−77.81	0.1148	2	11.515	0.1180
3	9.0871	8.9286	2.136	−77.03	0.1149			
4	9.093	9.2307	2.128	−76.75	0.1154	3	9.3172	0.1185
5	9.1248	9.2307	2.128	−76.75	0.1154	4	9.3556	0.1185
**It.**	**Calcul. Freq (Hz)**	**Meas. Freq (Hz)**	**Re (Ohm)**	**Im (Ohm)**	**Conduct. (mS/cm)**	**It**	**Calcul. Freq (Hz)**	**Conduct. (mS/cm)**
1		500	235	−123	0.9397	1		0.9515
2	69,132	68,571.4	187	−6.25	1.3303	2	69,995	1.3489
3	97,569	96,000	186.4	−5.94	1.339			
4	98,244	100,000	185.9	−5.92	1.34	3	98,923	1.3566
5	98,491	100,000	185.9	−5.92	1.34	4	99,483	1.3566

**Figure 9:**
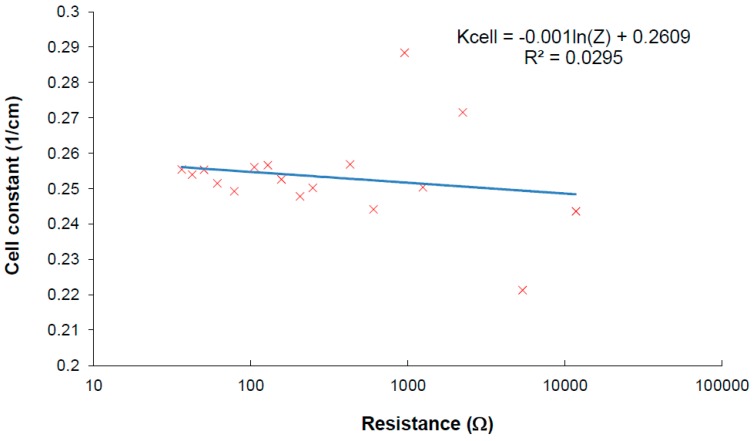
Verification of cell constant deviation when the developed method is used for measurement.

The algorithm of measurement was applied on several samples of solutions. After every step the new frequency was calculated from correction Equations (5) and with the cell constant treatment at two modes. At the mode 1 the cell constant is corrected by Equation (6), at mode 2 the average cell constant (from Figure 6 or Figure 9) is used. Some examples for two different samples are presented in Table 1; a KCl solution with conductivity 0.12 mS/cm and 1.3 mS/cm. The iteration process goes very quickly to a value close to the frequency at local maxima. Only four steps at the mode 1 and three steps at the mode 2 were achieved to reach the highest precise value of the measured conductivity in our experiment. The impedance analyzer which was used does not have a very fine tunable generator, so only varied steps not at exact frequency could be used. For this reason, higher accuracy cannot be obtained with the impedance analyzer. Probably more steps could be done to better approximate to the real specific conductivity and to obtain higher accuracy if a fine tunable generator would be applied in the impedance measurement.

The mode 2 is faster with similar results. It can be the reason of better approximation to the real value of conductivity. It appears that cell constant corrected within measured impedance does not increase the accuracy of the measurement. The accuracy is definitely determined by correction frequency characteristics (Figure 6) and it depends on how precisely they are obtained in the characterization process of the electrode.

The verification of the method was performed using reverse calculation of the cell constant from measured results. The calibrating results are plotted in Figure 9, where the cell constant changes a little within the impedance measurement taken at the frequency at local maxima. Linear regression of the measured results shows the parameters for Equation (6). There are some values with higher deviation which can be explained as error of value determination going from insufficiently calibrated samples used in measurements. The plot shows that frequency iterative tuning within finding real specific conductivity increases the accuracy of the measurement at a wide range because the dependence of the cell constant presented in Figure 4 was minimized by the method.

## 5. Conclusions

The suggested method creates a tool how to process data easily and improve accuracy of the specific conductivity measurement of electrolytes at wide range with IDEs. The results were confirmed on electrolytes with ordinary redox pairs usually used in IDEs applications. The results show that high accuracy of the measurement with IDEs can be achieved using iterative approximation to real specific conductivity by tuning frequency according to determined equation characterizing the IDEs behavior. The methods can involve many steps and need calculation ability; therefore, the method is suitable for microcontroller applications and the measurements can be taken automatically to reduce measuring time. The fine tunable oscillator should be used to reach more precise measurements. The elimination of the cell replacement with the maintenance of the accuracy at the wide range of specific conductivity is the most important result of our experiments.
